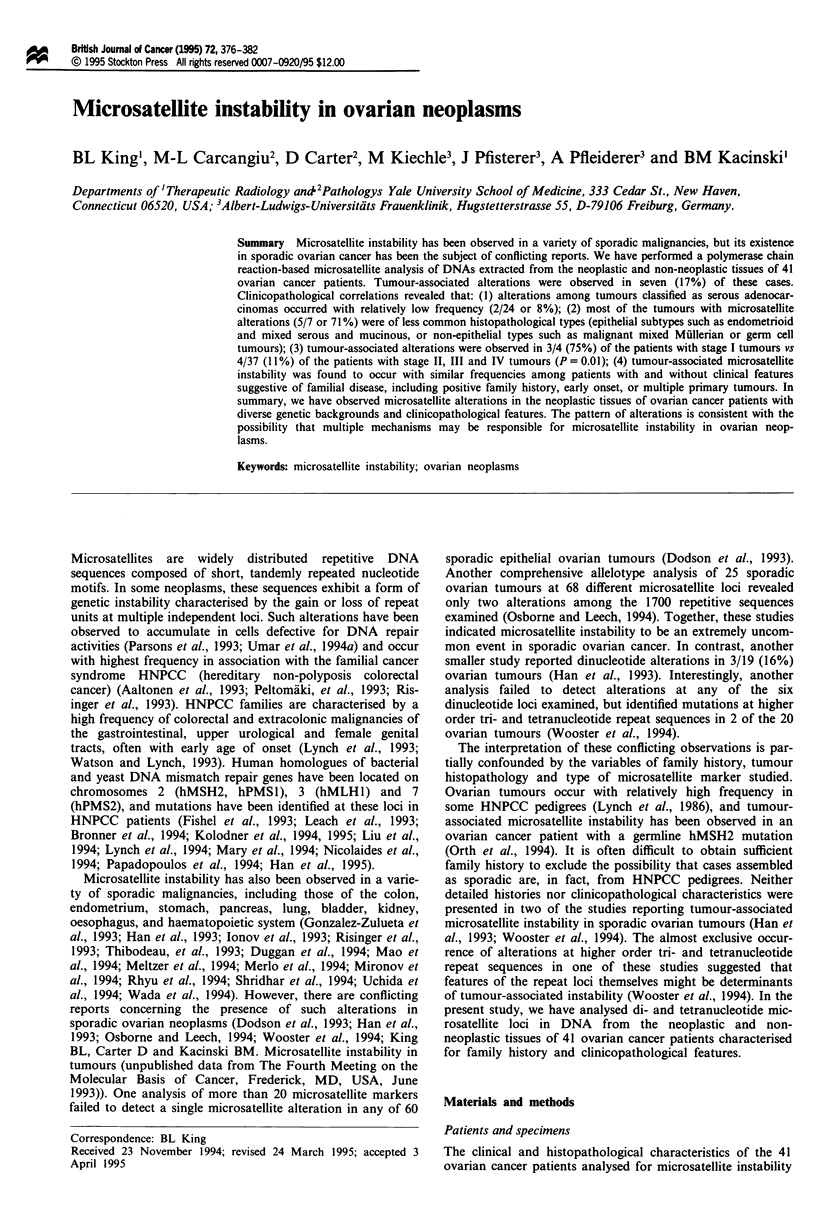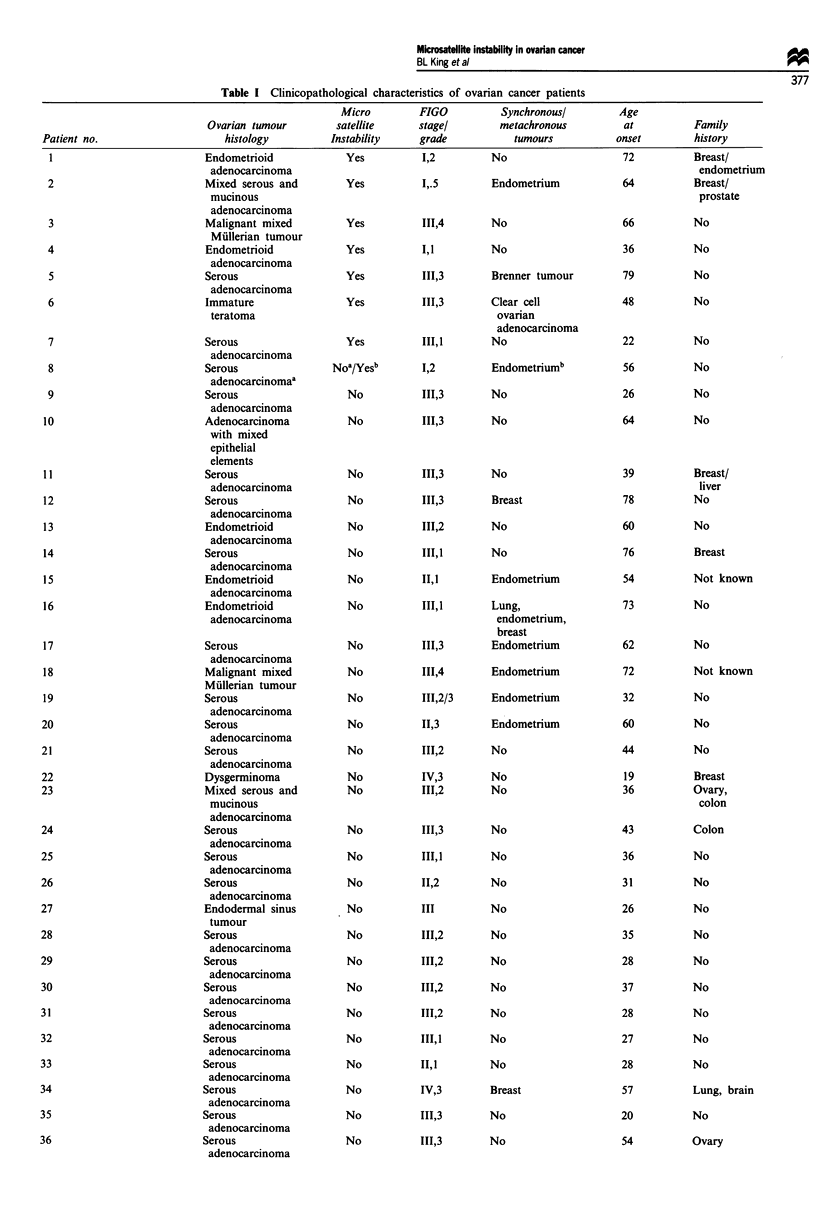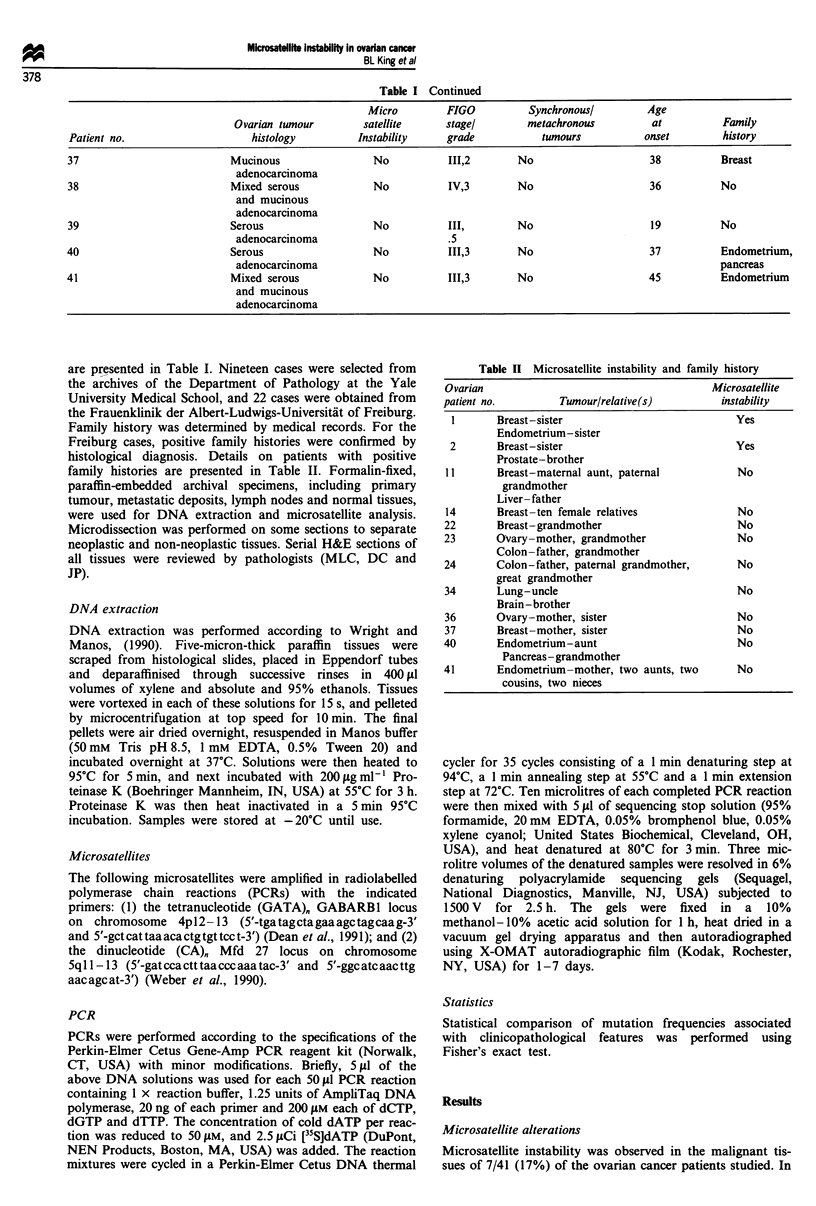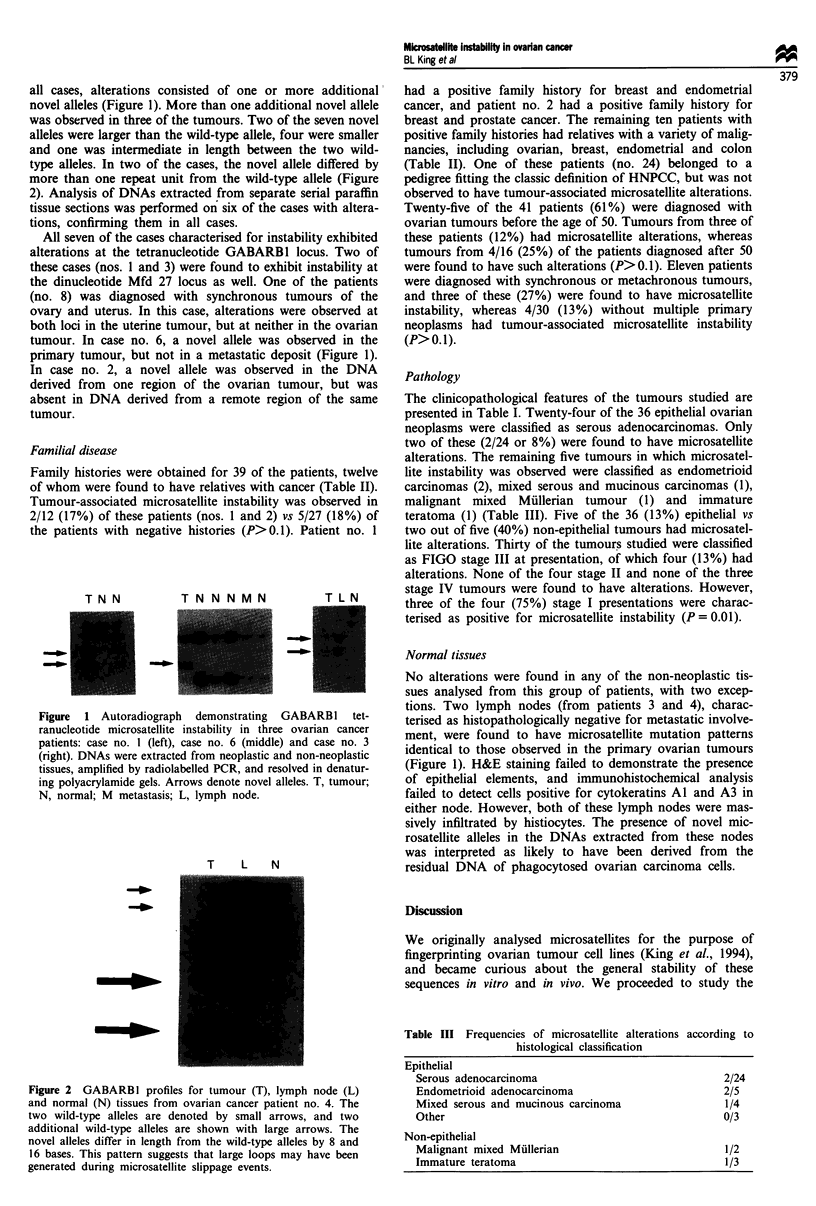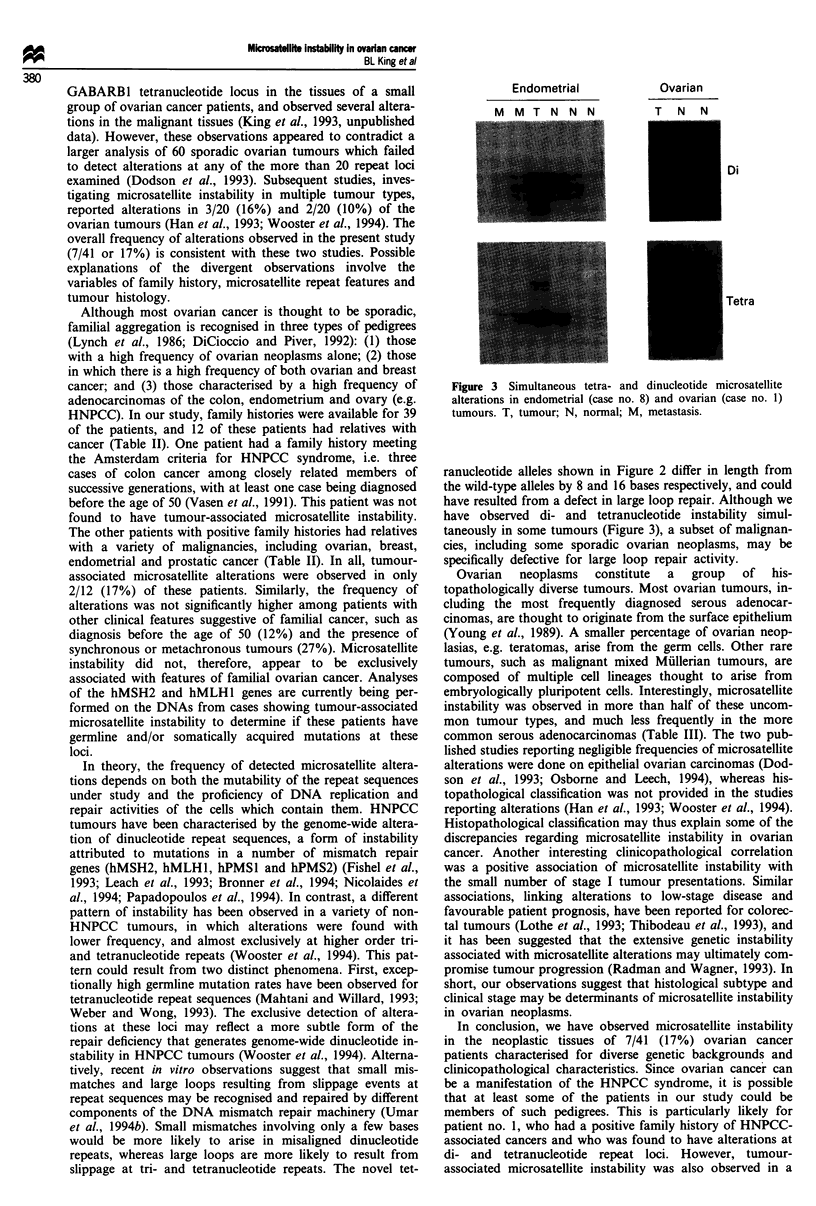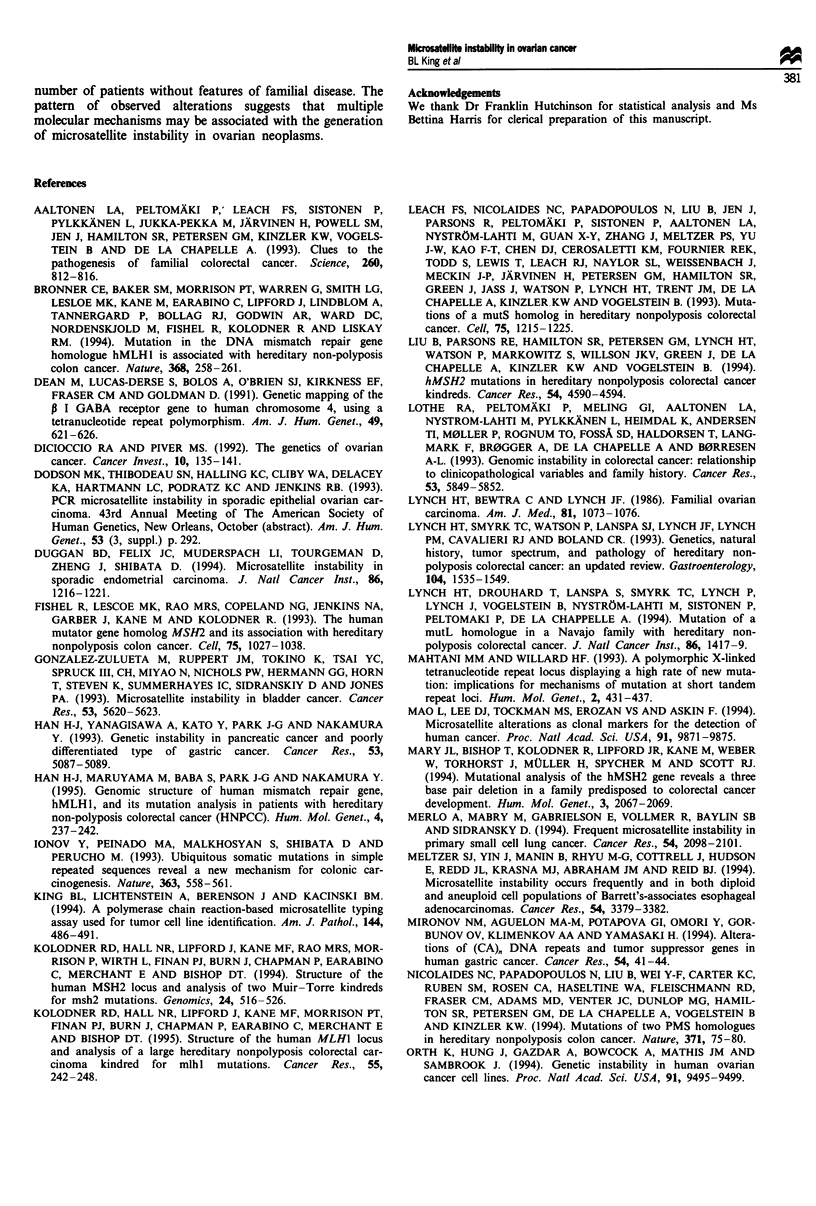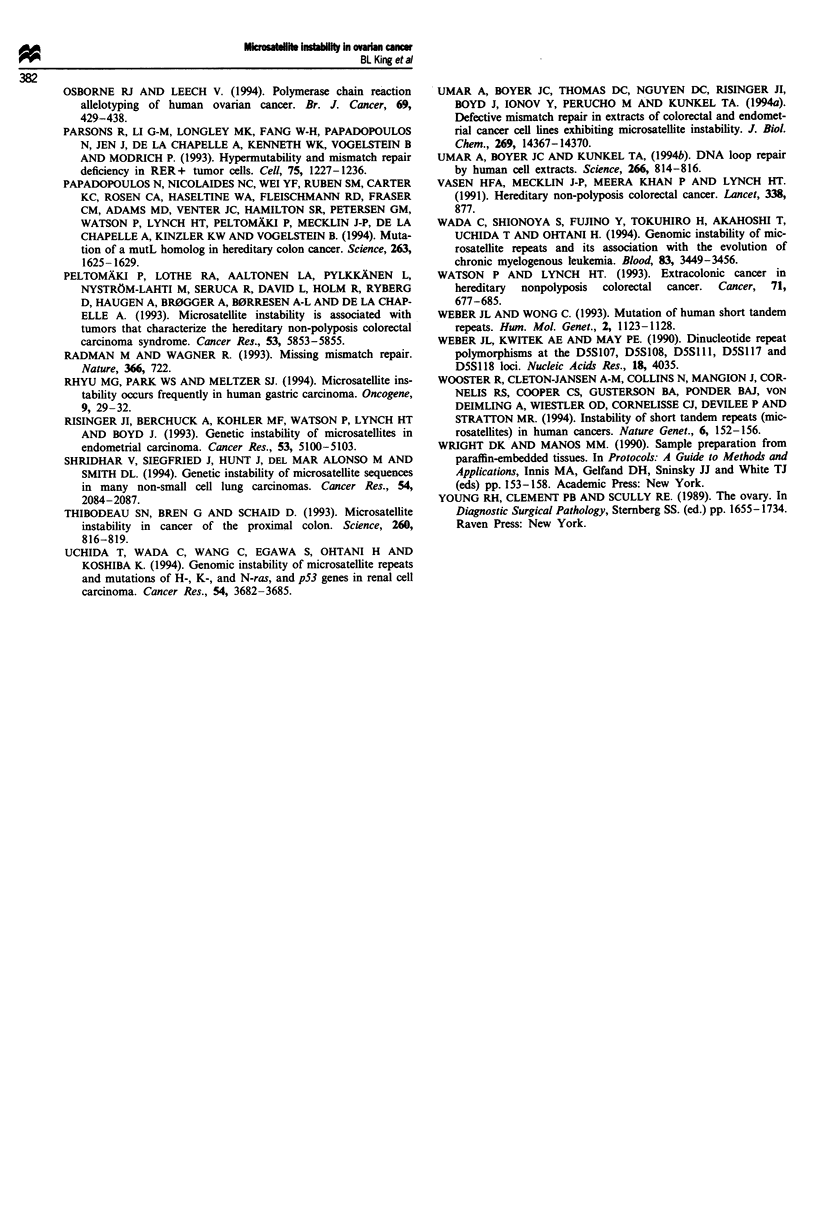# Microsatellite instability in ovarian neoplasms.

**DOI:** 10.1038/bjc.1995.341

**Published:** 1995-08

**Authors:** B. L. King, M. L. Carcangiu, D. Carter, M. Kiechle, J. Pfisterer, A. Pfleiderer, B. M. Kacinski

**Affiliations:** Department of Therapeutic Radiology, Yale University School of Medicine, New Haven, Connecticut 06520, USA.

## Abstract

**Images:**


British Journal of Cancer (1995) 72, 376-382
© 1995 Stockton Press All rights reserved 0007-0920/95 $12.00

### Microsatellite instability in ovarian neoplasms

BL King[1], M-L Carcangiu[2], D Carter[2], M Kiechle[3], J Pfisterer[3], A Pfleiderer[3] and BM Kacinski[1]

*Departments of [1]Therapeutic Radiology and [2]Pathologys Yale University School of Medicine, 333 Cedar St., New Haven, Connecticut 06520, USA; [3]Albert-Ludwigs-Universitäts Frauenklinik, Hugstetterstrasse 55, D-79106 Freiburg, Germany.*

**Summary** Microsatellite instability has been observed in a variety of sporadic malignancies, but its existence in sporadic ovarian cancer has been the subject of conflicting reports. We have performed a polymerase chain reaction-based microsatellite analysis of DNAs extracted from the neoplastic and non-neoplastic tissues of 41 ovarian cancer patients. Tumour-associated alterations were observed in seven (17%) of these cases. Clinicopathological correlations revealed that: (1) alterations among tumours classified as serous adenocarcinomas occurred with relatively low frequency (2/24 or 8%); (2) most of the tumours with microsatellite alterations (5/7 or 71%) were of less common histopathological types (epithelial subtypes such as endometrioid and mixed serous and mucinous, or non-epithelial types such as malignant mixed Müllerian or germ cell tumours); (3) tumour-associated alterations were observed in 3/4 (75%) of the patients with stage I tumours *vs* 4/37 (11%) of the patients with stage II, III and IV tumours ($P = 0.01$); (4) tumour-associated microsatellite instability was found to occur with similar frequencies among patients with and without clinical features suggestive of familial disease, including positive family history, early onset, or multiple primary tumours. In summary, we have observed microsatellite alterations in the neoplastic tissues of ovarian cancer patients with diverse genetic backgrounds and clinicopathological features. The pattern of alterations is consistent with the possibility that multiple mechanisms may be responsible for microsatellite instability in ovarian neoplasms.

**Keywords:** microsatellite instability; ovarian neoplasms

Microsatellites are widely distributed repetitive DNA sequences composed of short, tandemly repeated nucleotide motifs. In some neoplasms, these sequences exhibit a form of genetic instability characterised by the gain or loss of repeat units at multiple independent loci. Such alterations have been observed to accumulate in cells defective for DNA repair activities (Parsons *et al.*, 1993; Umar *et al.*, 1994*a*) and occur with highest frequency in association with the familial cancer syndrome HNPCC (hereditary non-polyposis colorectal cancer) (Aaltonen *et al.*, 1993; Peltomäki, *et al.*, 1993; Risinger *et al.*, 1993). HNPCC families are characterised by a high frequency of colorectal and extracolonic malignancies of the gastrointestinal, upper urological and female genital tracts, often with early age of onset (Lynch *et al.*, 1993; Watson and Lynch, 1993). Human homologues of bacterial and yeast DNA mismatch repair genes have been located on chromosomes 2 (hMSH2, hPMS1), 3 (hMLH1) and 7 (hPMS2), and mutations have been identified at these loci in HNPCC patients (Fishel *et al.*, 1993; Leach *et al.*, 1993; Bronner *et al.*, 1994; Kolodner *et al.*, 1994, 1995; Liu *et al.*, 1994; Lynch *et al.*, 1994; Mary *et al.*, 1994; Nicolaides *et al.*, 1994; Papadopoulos *et al.*, 1994; Han *et al.*, 1995).

Microsatellite instability has also been observed in a variety of sporadic malignancies, including those of the colon, endometrium, stomach, pancreas, lung, bladder, kidney, oesophagus, and haematopoietic system (Gonzalez-Zulueta *et al.*, 1993; Han *et al.*, 1993; Ionov *et al.*, 1993; Risinger *et al.*, 1993; Thibodeau, *et al.*, 1993; Duggan *et al.*, 1994; Mao *et al.*, 1994; Meltzer *et al.*, 1994; Merlo *et al.*, 1994; Mironov *et al.*, 1994; Rhyu *et al.*, 1994; Shridhar *et al.*, 1994; Uchida *et al.*, 1994; Wada *et al.*, 1994). However, there are conflicting reports concerning the presence of such alterations in sporadic ovarian neoplasms (Dodson *et al.*, 1993; Han *et al.*, 1993; Osborne and Leech, 1994; Wooster *et al.*, 1994; King BL, Carter D and Kacinski BM. Microsatellite instability in tumours (unpublished data from The Fourth Meeting on the Molecular Basis of Cancer, Frederick, MD, USA, June 1993)). One analysis of more than 20 microsatellite markers failed to detect a single microsatellite alteration in any of 60 sporadic epithelial ovarian tumours (Dodson *et al.*, 1993). Another comprehensive allelotype analysis of 25 sporadic ovarian tumours at 68 different microsatellite loci revealed only two alterations among the 1700 repetitive sequences examined (Osborne and Leech, 1994). Together, these studies indicated microsatellite instability to be an extremely uncommon event in sporadic ovarian cancer. In contrast, another smaller study reported dinucleotide alterations in 3/19 (16%) ovarian tumours (Han *et al.*, 1993). Interestingly, another analysis failed to detect alterations at any of the six dinucleotide loci examined, but identified mutations at higher order tri- and tetranucleotide repeat sequences in 2 of the 20 ovarian tumours (Wooster *et al.*, 1994).

The interpretation of these conflicting observations is partially confounded by the variables of family history, tumour histopathology and type of microsatellite marker studied. Ovarian tumours occur with relatively high frequency in some HNPCC pedigrees (Lynch *et al.*, 1986), and tumour-associated microsatellite instability has been observed in an ovarian cancer patient with a germline hMSH2 mutation (Orth *et al.*, 1994). It is often difficult to obtain sufficient family history to exclude the possibility that cases assembled as sporadic are, in fact, from HNPCC pedigrees. Neither detailed histories nor clinicopathological characteristics were presented in two of the studies reporting tumour-associated microsatellite instability in sporadic ovarian tumours (Han *et al.*, 1993; Wooster *et al.*, 1994). The almost exclusive occurrence of alterations at higher order tri- and tetranucleotide repeat sequences in one of these studies suggested that features of the repeat loci themselves might be determinants of tumour-associated instability (Wooster *et al.*, 1994). In the present study, we have analysed di- and tetranucleotide microsatellite loci in DNA from the neoplastic and non-neoplastic tissues of 41 ovarian cancer patients characterised for family history and clinicopathological features.

### Materials and methods

### *Patients and specimens*

The clinical and histopathological characteristics of the 41 ovarian cancer patients analysed for microsatellite instability

Correspondence: BL King
Received 23 November 1994; revised 24 March 1995; accepted 3 April 1995

**Table I** Clinicopathological characteristics of ovarian cancer patients

| *Patient no.* | *Ovarian tumour histology* | *Micro satellite Instability* | *FIGO stage/ grade* | *Synchronous/ metachronous tumours* | *Age at onset* | *Family history* |
|---|---|---|---|---|---|---|
| 1 | Endometrioid adenocarcinoma | Yes | I,2 | No | 72 | Breast/ endometrium |
| 2 | Mixed serous and mucinous adenocarcinoma | Yes | I,.5 | Endometrium | 64 | Breast/ prostate |
| 3 | Malignant mixed Müllerian tumour | Yes | III,4 | No | 66 | No |
| 4 | Endometrioid adenocarcinoma | Yes | I,1 | No | 36 | No |
| 5 | Serous adenocarcinoma | Yes | III,3 | Brenner tumour | 79 | No |
| 6 | Immature teratoma | Yes | III,3 | Clear cell ovarian adenocarcinoma | 48 | No |
| 7 | Serous adenocarcinoma | Yes | III,1 | No | 22 | No |
| 8 | Serous adenocarcinoma[a] | No[a]/Yes[b] | I,2 | Endometrium[b] | 56 | No |
| 9 | Serous adenocarcinoma | No | III,3 | No | 26 | No |
| 10 | Adenocarcinoma with mixed epithelial elements | No | III,3 | No | 64 | No |
| 11 | Serous adenocarcinoma | No | III,3 | No | 39 | Breast/ liver |
| 12 | Serous adenocarcinoma | No | III,3 | Breast | 78 | No |
| 13 | Endometrioid adenocarcinoma | No | III,2 | No | 60 | No |
| 14 | Serous adenocarcinoma | No | III,1 | No | 76 | Breast |
| 15 | Endometrioid adenocarcinoma | No | II,1 | Endometrium | 54 | Not known |
| 16 | Endometrioid adenocarcinoma | No | III,1 | Lung, endometrium, breast | 73 | No |
| 17 | Serous adenocarcinoma | No | III,3 | Endometrium | 62 | No |
| 18 | Malignant mixed Müllerian tumour | No | III,4 | Endometrium | 72 | Not known |
| 19 | Serous adenocarcinoma | No | III,2/3 | Endometrium | 32 | No |
| 20 | Serous adenocarcinoma | No | II,3 | Endometrium | 60 | No |
| 21 | Serous adenocarcinoma | No | III,2 | No | 44 | No |
| 22 | Dysgerminoma | No | IV,3 | No | 19 | Breast |
| 23 | Mixed serous and mucinous adenocarcinoma | No | III,2 | No | 36 | Ovary, colon |
| 24 | Serous adenocarcinoma | No | III,3 | No | 43 | Colon |
| 25 | Serous adenocarcinoma | No | III,1 | No | 36 | No |
| 26 | Serous adenocarcinoma | No | II,2 | No | 31 | No |
| 27 | Endodermal sinus tumour | No | III | No | 26 | No |
| 28 | Serous adenocarcinoma | No | III,2 | No | 35 | No |
| 29 | Serous adenocarcinoma | No | III,2 | No | 28 | No |
| 30 | Serous adenocarcinoma | No | III,2 | No | 37 | No |
| 31 | Serous adenocarcinoma | No | III,2 | No | 28 | No |
| 32 | Serous adenocarcinoma | No | III,1 | No | 27 | No |
| 33 | Serous adenocarcinoma | No | II,1 | No | 28 | No |
| 34 | Serous adenocarcinoma | No | IV,3 | Breast | 57 | Lung, brain |
| 35 | Serous adenocarcinoma | No | III,3 | No | 20 | No |
| 36 | Serous adenocarcinoma | No | III,3 | No | 54 | Ovary |

**Table I** Continued

| Patient no. | Ovarian tumour histology | Micro satellite Instability | FIGO stage/ grade | Synchronous/ metachronous tumours | Age at onset | Family history |
|---|---|---|---|---|---|---|
| 37 | Mucinous adenocarcinoma | No | III,2 | No | 38 | Breast |
| 38 | Mixed serous and mucinous adenocarcinoma | No | IV,3 | No | 36 | No |
| 39 | Serous adenocarcinoma | No | III, .5 | No | 19 | No |
| 40 | Serous adenocarcinoma | No | III,3 | No | 37 | Endometrium, pancreas |
| 41 | Mixed serous and mucinous adenocarcinoma | No | III,3 | No | 45 | Endometrium |

are presented in Table I. Nineteen cases were selected from the archives of the Department of Pathology at the Yale University Medical School, and 22 cases were obtained from the Frauenklinik der Albert-Ludwigs-Universität of Freiburg. Family history was determined by medical records. For the Freiburg cases, positive family histories were confirmed by histological diagnosis. Details on patients with positive family histories are presented in Table II. Formalin-fixed, paraffin-embedded archival specimens, including primary tumour, metastatic deposits, lymph nodes and normal tissues, were used for DNA extraction and microsatellite analysis. Microdissection was performed on some sections to separate neoplastic and non-neoplastic tissues. Serial H&E sections of all tissues were reviewed by pathologists (MLC, DC and JP).

*DNA extraction*

DNA extraction was performed according to Wright and Manos, (1990). Five-micron-thick paraffin tissues were scraped from histological slides, placed in Eppendorf tubes and deparaffinised through successive rinses in 400 $\mu$l volumes of xylene and absolute and 95% ethanols. Tissues were vortexed in each of these solutions for 15 s, and pelleted by microcentrifugation at top speed for 10 min. The final pellets were air dried overnight, resuspended in Manos buffer (50 mM Tris pH 8.5, 1 mM EDTA, 0.5% Tween 20) and incubated overnight at 37°C. Solutions were then heated to 95°C for 5 min, and next incubated with 200 $\mu$g ml$^{-1}$ Proteinase K (Boehringer Mannheim, IN, USA) at 55°C for 3 h. Proteinase K was then heat inactivated in a 5 min 95°C incubation. Samples were stored at $-20$°C until use.

*Microsatellites*

The following microsatellites were amplified in radiolabelled polymerase chain reactions (PCRs) with the indicated primers: (1) the tetranucleotide $(GATA)_n$ GABARB1 locus on chromosome 4p12–13 (5′-tgatagctagaaagctagcaag-3′ and 5′-gctcattaaacactgtgttcct-3′) (Dean *et al.*, 1991); and (2) the dinucleotide $(CA)_n$ Mfd 27 locus on chromosome 5q11–13 (5′-gatccacttaacccaaatac-3′ and 5′-ggcatcaacttg aacagcat-3′) (Weber *et al.*, 1990).

*PCR*

PCRs were performed according to the specifications of the Perkin-Elmer Cetus Gene-Amp PCR reagent kit (Norwalk, CT, USA) with minor modifications. Briefly, 5 $\mu$l of the above DNA solutions was used for each 50 $\mu$l PCR reaction containing 1 × reaction buffer, 1.25 units of AmpliTaq DNA polymerase, 20 ng of each primer and 200 $\mu$M each of dCTP, dGTP and dTTP. The concentration of cold dATP per reaction was reduced to 50 $\mu$M, and 2.5 $\mu$Ci [$^{35}$S]dATP (DuPont, NEN Products, Boston, MA, USA) was added. The reaction mixtures were cycled in a Perkin-Elmer Cetus DNA thermal cycler for 35 cycles consisting of a 1 min denaturing step at 94°C, a 1 min annealing step at 55°C and a 1 min extension step at 72°C. Ten microlitres of each completed PCR reaction were then mixed with 5 $\mu$l of sequencing stop solution (95% formamide, 20 mM EDTA, 0.05% bromphenol blue, 0.05% xylene cyanol; United States Biochemical, Cleveland, OH, USA), and heat denatured at 80°C for 3 min. Three microlitre volumes of the denatured samples were resolved in 6% denaturing polyacrylamide sequencing gels (Sequagel, National Diagnostics, Manville, NJ, USA) subjected to 1500 V for 2.5 h. The gels were fixed in a 10% methanol–10% acetic acid solution for 1 h, heat dried in a vacuum gel drying apparatus and then autoradiographed using X-OMAT autoradiographic film (Kodak, Rochester, NY, USA) for 1–7 days.

**Table II** Microsatellite instability and family history

| Ovarian patient no. | Tumour/relative(s) | Microsatellite instability |
|---|---|---|
| 1 | Breast–sister<br>Endometrium–sister | Yes |
| 2 | Breast–sister<br>Prostate–brother | Yes |
| 11 | Breast–maternal aunt, paternal grandmother<br>Liver–father | No |
| 14 | Breast–ten female relatives | No |
| 22 | Breast–grandmother | No |
| 23 | Ovary–mother, grandmother<br>Colon–father, grandmother | No |
| 24 | Colon–father, paternal grandmother, great grandmother | No |
| 34 | Lung–uncle<br>Brain–brother | No |
| 36 | Ovary–mother, sister | No |
| 37 | Breast–mother, sister | No |
| 40 | Endometrium–aunt<br>Pancreas–grandmother | No |
| 41 | Endometrium–mother, two aunts, two cousins, two nieces | No |

*Statistics*

Statistical comparison of mutation frequencies associated with clinicopathological features was performed using Fisher's exact test.

### Results

*Microsatellite alterations*

Microsatellite instability was observed in the malignant tissues of 7/41 (17%) of the ovarian cancer patients studied. In all cases, alterations consisted of one or more additional novel alleles (Figure 1). More than one additional novel allele was observed in three of the tumours. Two of the seven novel alleles were larger than the wild-type allele, four were smaller and one was intermediate in length between the two wild-type alleles. In two of the cases, the novel allele differed by more than one repeat unit from the wild-type allele (Figure 2). Analysis of DNAs extracted from separate serial paraffin tissue sections was performed on six of the cases with alterations, confirming them in all cases.

All seven of the cases characterised for instability exhibited alterations at the tetranucleotide GABARB1 locus. Two of these cases (nos. 1 and 3) were found to exhibit instability at the dinucleotide Mfd 27 locus as well. One of the patients (no. 8) was diagnosed with synchronous tumours of the ovary and uterus. In this case, alterations were observed at both loci in the uterine tumour, but at neither in the ovarian tumour. In case no. 6, a novel allele was observed in the primary tumour, but not in a metastatic deposit (Figure 1). In case no. 2, a novel allele was observed in the DNA derived from one region of the ovarian tumour, but was absent in DNA derived from a remote region of the same tumour.

*Familial disease*

Family histories were obtained for 39 of the patients, twelve of whom were found to have relatives with cancer (Table II). Tumour-associated microsatellite instability was observed in 2/12 (17%) of these patients (nos. 1 and 2) *vs* 5/27 (18%) of the patients with negative histories ($P > 0.1$). Patient no. 1 had a positive family history for breast and endometrial cancer, and patient no. 2 had a positive family history for breast and prostate cancer. The remaining ten patients with positive family histories had relatives with a variety of malignancies, including ovarian, breast, endometrial and colon (Table II). One of these patients (no. 24) belonged to a pedigree fitting the classic definition of HNPCC, but was not observed to have tumour-associated microsatellite alterations. Twenty-five of the 41 patients (61%) were diagnosed with ovarian tumours before the age of 50. Tumours from three of these patients (12%) had microsatellite alterations, whereas tumours from 4/16 (25%) of the patients diagnosed after 50 were found to have such alterations ($P > 0.1$). Eleven patients were diagnosed with synchronous or metachronous tumours, and three of these (27%) were found to have microsatellite instability, whereas 4/30 (13%) without multiple primary neoplasms had tumour-associated microsatellite instability ($P > 0.1$).

**Figure 1** Autoradiograph demonstrating GABARB1 tetranucleotide microsatellite instability in three ovarian cancer patients: case no. 1 (left), case no. 6 (middle) and case no. 3 (right). DNAs were extracted from neoplastic and non-neoplastic tissues, amplified by radiolabelled PCR, and resolved in denaturing polyacrylamide gels. Arrows denote novel alleles. T, tumour; N, normal; M metastasis; L, lymph node.

**Figure 2** GABARB1 profiles for tumour (T), lymph node (L) and normal (N) tissues from ovarian cancer patient no. 4. The two wild-type alleles are denoted by small arrows, and two additional wild-type alleles are shown with large arrows. The novel alleles differ in length from the wild-type alleles by 8 and 16 bases. This pattern suggests that large loops may have been generated during microsatellite slippage events.

*Pathology*

The clinicopathological features of the tumours studied are presented in Table I. Twenty-four of the 36 epithelial ovarian neoplasms were classified as serous adenocarcinomas. Only two of these (2/24 or 8%) were found to have microsatellite alterations. The remaining five tumours in which microsatellite instability was observed were classified as endometrioid carcinomas (2), mixed serous and mucinous carcinomas (1), malignant mixed Müllerian tumour (1) and immature teratoma (1) (Table III). Five of the 36 (13%) epithelial *vs* two out of five (40%) non-epithelial tumours had microsatellite alterations. Thirty of the tumours studied were classified as FIGO stage III at presentation, of which four (13%) had alterations. None of the four stage II and none of the three stage IV tumours were found to have alterations. However, three of the four (75%) stage I presentations were characterised as positive for microsatellite instability ($P = 0.01$).

*Normal tissues*

No alterations were found in any of the non-neoplastic tissues analysed from this group of patients, with two exceptions. Two lymph nodes (from patients 3 and 4), characterised as histopathologically negative for metastatic involvement, were found to have microsatellite mutation patterns identical to those observed in the primary ovarian tumours (Figure 1). H&E staining failed to demonstrate the presence of epithelial elements, and immunohistochemical analysis failed to detect cells positive for cytokeratins A1 and A3 in either node. However, both of these lymph nodes were massively infiltrated by histiocytes. The presence of novel microsatellite alleles in the DNAs extracted from these nodes was interpreted as likely to have been derived from the residual DNA of phagocytosed ovarian carcinoma cells.

### Discussion

We originally analysed microsatellites for the purpose of fingerprinting ovarian tumour cell lines (King *et al.*, 1994), and became curious about the general stability of these sequences *in vitro* and *in vivo*. We proceeded to study the

**Table III** Frequencies of microsatellite alterations according to histological classification

| | |
|---|---|
| Epithelial | |
| Serous adenocarcinoma | 2/24 |
| Endometrioid adenocarcinoma | 2/5 |
| Mixed serous and mucinous carcinoma | 1/4 |
| Other | 0/3 |
| Non-epithelial | |
| Malignant mixed Müllerian | 1/2 |
| Immature teratoma | 1/3 |

GABARB1 tetranucleotide locus in the tissues of a small group of ovarian cancer patients, and observed several alterations in the malignant tissues (King *et al.*, 1993, unpublished data). However, these observations appeared to contradict a larger analysis of 60 sporadic ovarian tumours which failed to detect alterations at any of the more than 20 repeat loci examined (Dodson *et al.*, 1993). Subsequent studies, investigating microsatellite instability in multiple tumour types, reported alterations in 3/20 (16%) and 2/20 (10%) of the ovarian tumours (Han *et al.*, 1993; Wooster *et al.*, 1994). The overall frequency of alterations observed in the present study (7/41 or 17%) is consistent with these two studies. Possible explanations of the divergent observations involve the variables of family history, microsatellite repeat features and tumour histology.

Although most ovarian cancer is thought to be sporadic, familial aggregation is recognised in three types of pedigrees (Lynch *et al.*, 1986; DiCioccio and Piver, 1992): (1) those with a high frequency of ovarian neoplasms alone; (2) those in which there is a high frequency of both ovarian and breast cancer; and (3) those characterised by a high frequency of adenocarcinomas of the colon, endometrium and ovary (e.g. HNPCC). In our study, family histories were available for 39 of the patients, and 12 of these patients had relatives with cancer (Table II). One patient had a family history meeting the Amsterdam criteria for HNPCC syndrome, i.e. three cases of colon cancer among closely related members of successive generations, with at least one case being diagnosed before the age of 50 (Vasen *et al.*, 1991). This patient was not found to have tumour-associated microsatellite instability. The other patients with positive family histories had relatives with a variety of malignancies, including ovarian, breast, endometrial and prostatic cancer (Table II). In all, tumour-associated microsatellite alterations were observed in only 2/12 (17%) of these patients. Similarly, the frequency of alterations was not significantly higher among patients with other clinical features suggestive of familial cancer, such as diagnosis before the age of 50 (12%) and the presence of synchronous or metachronous tumours (27%). Microsatellite instability did not, therefore, appear to be exclusively associated with features of familial ovarian cancer. Analyses of the hMSH2 and hMLH1 genes are currently being performed on the DNAs from cases showing tumour-associated microsatellite instability to determine if these patients have germline and/or somatically acquired mutations at these loci.

In theory, the frequency of detected microsatellite alterations depends on both the mutability of the repeat sequences under study and the proficiency of DNA replication and repair activities of the cells which contain them. HNPCC tumours have been characterised by the genome-wide alteration of dinucleotide repeat sequences, a form of instability attributed to mutations in a number of mismatch repair genes (hMSH2, hMLH1, hPMS1 and hPMS2) (Fishel *et al.*, 1993; Leach *et al.*, 1993; Bronner *et al.*, 1994; Nicolaides *et al.*, 1994; Papadopoulos *et al.*, 1994). In contrast, a different pattern of instability has been observed in a variety of non-HNPCC tumours, in which alterations were found with lower frequency, and almost exclusively at higher order tri- and tetranucleotide repeats (Wooster *et al.*, 1994). This pattern could result from two distinct phenomena. First, exceptionally high germline mutation rates have been observed for tetranucleotide repeat sequences (Mahtani and Willard, 1993; Weber and Wong, 1993). The exclusive detection of alterations at these loci may reflect a more subtle form of the repair deficiency that generates genome-wide dinucleotide instability in HNPCC tumours (Wooster *et al.*, 1994). Alternatively, recent *in vitro* observations suggest that small mismatches and large loops resulting from slippage events at repeat sequences may be recognised and repaired by different components of the DNA mismatch repair machinery (Umar *et al.*, 1994*b*). Small mismatches involving only a few bases would be more likely to arise in misaligned dinucleotide repeats, whereas large loops are more likely to result from slippage at tri- and tetranucleotide repeats. The novel tetranucleotide alleles shown in Figure 2 differ in length from the wild-type alleles by 8 and 16 bases respectively, and could have resulted from a defect in large loop repair. Although we have observed di- and tetranucleotide instability simultaneously in some tumours (Figure 3), a subset of malignancies, including some sporadic ovarian neoplasms, may be specifically defective for large loop repair activity.

**Figure 3** Simultaneous tetra- and dinucleotide microsatellite alterations in endometrial (case no. 8) and ovarian (case no. 1) tumours. T, tumour; N, normal; M, metastasis.

Ovarian neoplasms constitute a group of histopathologically diverse tumours. Most ovarian tumours, including the most frequently diagnosed serous adenocarcinomas, are thought to originate from the surface epithelium (Young *et al.*, 1989). A smaller percentage of ovarian neoplasias, e.g. teratomas, arise from the germ cells. Other rare tumours, such as malignant mixed Müllerian tumours, are composed of multiple cell lineages thought to arise from embryologically pluripotent cells. Interestingly, microsatellite instability was observed in more than half of these uncommon tumour types, and much less frequently in the more common serous adenocarcinomas (Table III). The two published studies reporting negligible frequencies of microsatellite alterations were done on epithelial ovarian carcinomas (Dodson *et al.*, 1993; Osborne and Leech, 1994), whereas histopathological classification was not provided in the studies reporting alterations (Han *et al.*, 1993; Wooster *et al.*, 1994). Histopathological classification may thus explain some of the discrepancies regarding microsatellite instability in ovarian cancer. Another interesting clinicopathological correlation was a positive association of microsatellite instability with the small number of stage I tumour presentations. Similar associations, linking alterations to low-stage disease and favourable patient prognosis, have been reported for colorectal tumours (Lothe *et al.*, 1993; Thibodeau *et al.*, 1993), and it has been suggested that the extensive genetic instability associated with microsatellite alterations may ultimately compromise tumour progression (Radman and Wagner, 1993). In short, our observations suggest that histological subtype and clinical stage may be determinants of microsatellite instability in ovarian neoplasms.

In conclusion, we have observed microsatellite instability in the neoplastic tissues of 7/41 (17%) ovarian cancer patients characterised for diverse genetic backgrounds and clinicopathological characteristics. Since ovarian cancer can be a manifestation of the HNPCC syndrome, it is possible that at least some of the patients in our study could be members of such pedigrees. This is particularly likely for patient no. 1, who had a positive family history of HNPCC-associated cancers and who was found to have alterations at di- and tetranucleotide repeat loci. However, tumour-associated microsatellite instability was also observed in a number of patients without features of familial disease. The pattern of observed alterations suggests that multiple molecular mechanisms may be associated with the generation of microsatellite instability in ovarian neoplasms.

**Acknowledgements**
We thank Dr Franklin Hutchinson for statistical analysis and Ms Bettina Harris for clerical preparation of this manuscript.

**References**

AALTONEN LA, PELTOMÄKI P,' LEACH FS, SISTONEN P, PYLKKÄNEN L, JUKKA-PEKKA M, JÄRVINEN H, POWELL SM, JEN J, HAMILTON SR, PETERSEN GM, KINZLER KW, VOGELSTEIN B AND DE LA CHAPELLE A. (1993). Clues to the pathogenesis of familial colorectal cancer. *Science*, **260**, 812–816.

BRONNER CE, BAKER SM, MORRISON PT, WARREN G, SMITH LG, LESLOE MK, KANE M, EARABINO C, LIPFORD J, LINDBLOM A, TANNERGARD P, BOLLAG RJ, GODWIN AR, WARD DC, NORDENSKJOLD M, FISHEL R, KOLODNER R AND LISKAY RM. (1994). Mutation in the DNA mismatch repair gene homologue hMLH1 is associated with hereditary non-polyposis colon cancer. *Nature*, **368**, 258–261.

DEAN M, LUCAS-DERSE S, BOLOS A, O'BRIEN SJ, KIRKNESS EF, FRASER CM AND GOLDMAN D. (1991). Genetic mapping of the β I GABA receptor gene to human chromosome 4, using a tetranucleotide repeat polymorphism. *Am. J. Hum. Genet.*, **49**, 621–626.

DICIOCCIO RA AND PIVER MS. (1992). The genetics of ovarian cancer. *Cancer Invest.*, **10**, 135–141.

DODSON MK, THIBODEAU SN, HALLING KC, CLIBY WA, DELACEY KA, HARTMANN LC, PODRATZ KC AND JENKINS RB. (1993). PCR microsatellite instability in sporadic epithelial ovarian carcinoma. 43rd Annual Meeting of The American Society of Human Genetics, New Orleans, October (abstract). *Am. J. Hum. Genet.*, **53** (3, suppl.) p. 292.

DUGGAN BD, FELIX JC, MUDERSPACH LI, TOURGEMAN D, ZHENG J, SHIBATA D. (1994). Microsatellite instability in sporadic endometrial carcinoma. *J. Natl Cancer Inst.*, **86**, 1216–1221.

FISHEL R, LESCOE MK, RAO MRS, COPELAND NG, JENKINS NA, GARBER J, KANE M AND KOLODNER R. (1993). The human mutator gene homolog *MSH2* and its association with hereditary nonpolyposis colon cancer. *Cell*, **75**, 1027–1038.

GONZALEZ-ZULUETA M, RUPPERT JM, TOKINO K, TSAI YC, SPRUCK III, CH, MIYAO N, NICHOLS PW, HERMANN GG, HORN T, STEVEN K, SUMMERHAYES IC, SIDRANSKIY D AND JONES PA. (1993). Microsatellite instability in bladder cancer. *Cancer Res.*, **53**, 5620–5623.

HAN H-J, YANAGISAWA A, KATO Y, PARK J-G AND NAKAMURA Y. (1993). Genetic instability in pancreatic cancer and poorly differentiated type of gastric cancer. *Cancer Res.*, **53**, 5087–5089.

HAN H-J, MARUYAMA M, BABA S, PARK J-G AND NAKAMURA Y. (1995). Genomic structure of human mismatch repair gene, hMLH1, and its mutation analysis in patients with hereditary non-polyposis colorectal cancer (HNPCC). *Hum. Mol. Genet.*, **4**, 237–242.

IONOV Y, PEINADO MA, MALKHOSYAN S, SHIBATA D AND PERUCHO M. (1993). Ubiquitous somatic mutations in simple repeated sequences reveal a new mechanism for colonic carcinogenesis. *Nature*, **363**, 558–561.

KING BL, LICHTENSTEIN A, BERENSON J AND KACINSKI BM. (1994). A polymerase chain reaction-based microsatellite typing assay used for tumor cell line identification. *Am. J. Pathol.*, **144**, 486–491.

KOLODNER RD, HALL NR, LIPFORD J, KANE MF, RAO MRS, MORRISON P, WIRTH L, FINAN PJ, BURN J, CHAPMAN P, EARABINO C, MERCHANT E AND BISHOP DT. (1994). Structure of the human MSH2 locus and analysis of two Muir–Torre kindreds for msh2 mutations. *Genomics*, **24**, 516–526.

KOLODNER RD, HALL NR, LIPFORD J, KANE MF, MORRISON PT, FINAN PJ, BURN J, CHAPMAN P, EARABINO C, MERCHANT E AND BISHOP DT. (1995). Structure of the human *MLH*1 locus and analysis of a large hereditary nonpolyposis colorectal carcinoma kindred for mlh1 mutations. *Cancer Res.*, **55**, 242–248.

LEACH FS, NICOLAIDES NC, PAPADOPOULOS N, LIU B, JEN J, PARSONS R, PELTOMÄKI P, SISTONEN P, AALTONEN LA, NYSTRÖM-LAHTI M, GUAN X-Y, ZHANG J, MELTZER PS, YU J-W, KAO F-T, CHEN DJ, CEROSALETTI KM, FOURNIER REK, TODD S, LEWIS T, LEACH RJ, NAYLOR SL, WEISSENBACH J, MECKIN J-P, JÄRVINEN H, PETERSEN GM, HAMILTON SR, GREEN J, JASS J, WATSON P, LYNCH HT, TRENT JM, DE LA CHAPELLE A, KINZLER KW AND VOGELSTEIN B. (1993). Mutations of a mutS homolog in hereditary nonpolyposis colorectal cancer. *Cell*, **75**, 1215–1225.

LIU B, PARSONS RE, HAMILTON SR, PETERSEN GM, LYNCH HT, WATSON P, MARKOWITZ S, WILLSON JKV, GREEN J, DE LA CHAPELLE A, KINZLER KW AND VOGELSTEIN B. (1994). *hMSH2* mutations in hereditary nonpolyposis colorectal cancer kindreds. *Cancer Res.*, **54**, 4590–4594.

LOTHE RA, PELTOMÄKI P, MELING GI, AALTONEN LA, NYSTROM-LAHTI M, PYLKKÄNEN L, HEIMDAL K, ANDERSEN TI, MØLLER P, ROGNUM TO, FOSSÅ SD, HALDORSEN T, LANGMARK F, BRØGGER A, DE LA CHAPELLE A AND BØRRESEN A-L. (1993). Genomic instability in colorectal cancer: relationship to clinicopathological variables and family history. *Cancer Res.*, **53**, 5849–5852.

LYNCH HT, BEWTRA C AND LYNCH JF. (1986). Familial ovarian carcinoma. *Am. J. Med.*, **81**, 1073–1076.

LYNCH HT, SMYRK TC, WATSON P, LANSPA SJ, LYNCH JF, LYNCH PM, CAVALIERI RJ AND BOLAND CR. (1993). Genetics, natural history, tumor spectrum, and pathology of hereditary nonpolyposis colorectal cancer: an updated review. *Gastroenterology*, **104**, 1535–1549.

LYNCH HT, DROUHARD T, LANSPA S, SMYRK TC, LYNCH P, LYNCH J, VOGELSTEIN B, NYSTRÖM-LAHTI M, SISTONEN P, PELTOMAKI P, DE LA CHAPPELLE A. (1994). Mutation of a mutL homologue in a Navajo family with hereditary nonpolyposis colorectal cancer. *J. Natl Cancer Inst.*, **86**, 1417–9.

MAHTANI MM AND WILLARD HF. (1993). A polymorphic X-linked tetranucleotide repeat locus displaying a high rate of new mutation: implications for mechanisms of mutation at short tandem repeat loci. *Hum. Mol. Genet.*, **2**, 431–437.

MAO L, LEE DJ, TOCKMAN MS, EROZAN VS AND ASKIN F. (1994). Microsatellite alterations as clonal markers for the detection of human cancer. *Proc. Natl Acad. Sci. USA*, **91**, 9871–9875.

MARY JL, BISHOP T, KOLODNER R, LIPFORD JR, KANE M, WEBER W, TORHORST J, MÜLLER H, SPYCHER M AND SCOTT RJ. (1994). Mutational analysis of the hMSH2 gene reveals a three base pair deletion in a family predisposed to colorectal cancer development. *Hum. Mol. Genet.*, **3**, 2067–2069.

MERLO A, MABRY M, GABRIELSON E, VOLLMER R, BAYLIN SB AND SIDRANSKY D. (1994). Frequent microsatellite instability in primary small cell lung cancer. *Cancer Res.*, **54**, 2098–2101.

MELTZER SJ, YIN J, MANIN B, RHYU M-G, COTTRELL J, HUDSON E, REDD JL, KRASNA MJ, ABRAHAM JM AND REID BJ. (1994). Microsatellite instability occurs frequently and in both diploid and aneuploid cell populations of Barrett's-associates esophageal adenocarcinomas. *Cancer Res.*, **54**, 3379–3382.

MIRONOV NM, AGUELON MA-M, POTAPOVA GI, OMORI Y, GORBUNOV OV, KLIMENKOV AA AND YAMASAKI H. (1994). Alterations of $(CA)_n$ DNA repeats and tumor suppressor genes in human gastric cancer. *Cancer Res.*, **54**, 41–44.

NICOLAIDES NC, PAPADOPOULOS N, LIU B, WEI Y-F, CARTER KC, RUBEN SM, ROSEN CA, HASELTINE WA, FLEISCHMANN RD, FRASER CM, ADAMS MD, VENTER JC, DUNLOP MG, HAMILTON SR, PETERSEN GM, DE LA CHAPELLE A, VOGELSTEIN B AND KINZLER KW. (1994). Mutations of two PMS homologues in hereditary nonpolyposis colon cancer. *Nature*, **371**, 75–80.

ORTH K, HUNG J, GAZDAR A, BOWCOCK A, MATHIS JM AND SAMBROOK J. (1994). Genetic instability in human ovarian cancer cell lines. *Proc. Natl Acad. Sci. USA*, **91**, 9495–9499.

OSBORNE RJ AND LEECH V. (1994). Polymerase chain reaction allelotyping of human ovarian cancer. *Br. J. Cancer*, **69**, 429–438.

PARSONS R, LI G-M, LONGLEY MK, FANG W-H, PAPADOPOULOS N, JEN J, DE LA CHAPELLE A, KENNETH WK, VOGELSTEIN B AND MODRICH P. (1993). Hypermutability and mismatch repair deficiency in RER+ tumor cells. *Cell*, **75**, 1227–1236.

PAPADOPOULOS N, NICOLAIDES NC, WEI YF, RUBEN SM, CARTER KC, ROSEN CA, HASELTINE WA, FLEISCHMANN RD, FRASER CM, ADAMS MD, VENTER JC, HAMILTON SR, PETERSEN GM, WATSON P, LYNCH HT, PELTOMÄKI P, MECKLIN J-P, DE LA CHAPELLE A, KINZLER KW AND VOGELSTEIN B. (1994). Mutation of a mutL homolog in hereditary colon cancer. *Science*, **263**, 1625–1629.

PELTOMÄKI P, LOTHE RA, AALTONEN LA, PYLKKÄNEN L, NYSTRÖM-LAHTI M, SERUCA R, DAVID L, HOLM R, RYBERG D, HAUGEN A, BRØGGER A, BØRRESEN A-L AND DE LA CHAPELLE A. (1993). Microsatellite instability is associated with tumors that characterize the hereditary non-polyposis colorectal carcinoma syndrome. *Cancer Res.*, **53**, 5853–5855.

RADMAN M AND WAGNER R. (1993). Missing mismatch repair. *Nature*, **366**, 722.

RHYU MG, PARK WS AND MELTZER SJ. (1994). Microsatellite instability occurs frequently in human gastric carcinoma. *Oncogene*, **9**, 29–32.

RISINGER JI, BERCHUCK A, KOHLER MF, WATSON P, LYNCH HT AND BOYD J. (1993). Genetic instability of microsatellites in endometrial carcinoma. *Cancer Res.*, **53**, 5100–5103.

SHRIDHAR V, SIEGFRIED J, HUNT J, DEL MAR ALONSO M AND SMITH DL. (1994). Genetic instability of microsatellite sequences in many non-small cell lung carcinomas. *Cancer Res.*, **54**, 2084–2087.

THIBODEAU SN, BREN G AND SCHAID D. (1993). Microsatellite instability in cancer of the proximal colon. *Science*, **260**, 816–819.

UCHIDA T, WADA C, WANG C, EGAWA S, OHTANI H AND KOSHIBA K. (1994). Genomic instability of microsatellite repeats and mutations of H-, K-, and N-*ras*, and *p53* genes in renal cell carcinoma. *Cancer Res.*, **54**, 3682–3685.

UMAR A, BOYER JC, THOMAS DC, NGUYEN DC, RISINGER JI, BOYD J, IONOV Y, PERUCHO M AND KUNKEL TA. (1994*a*). Defective mismatch repair in extracts of colorectal and endometrial cancer cell lines exhibiting microsatellite instability. *J. Biol. Chem.*, **269**, 14367–14370.

UMAR A, BOYER JC AND KUNKEL TA, (1994*b*). DNA loop repair by human cell extracts. *Science*, **266**, 814–816.

VASEN HFA, MECKLIN J-P, MEERA KHAN P AND LYNCH HT. (1991). Hereditary non-polyposis colorectal cancer. *Lancet*, **338**, 877.

WADA C, SHIONOYA S, FUJINO Y, TOKUHIRO H, AKAHOSHI T, UCHIDA T AND OHTANI H. (1994). Genomic instability of microsatellite repeats and its association with the evolution of chronic myelogenous leukemia. *Blood*, **83**, 3449–3456.

WATSON P AND LYNCH HT. (1993). Extracolonic cancer in hereditary nonpolyposis colorectal cancer. *Cancer*, **71**, 677–685.

WEBER JL AND WONG C. (1993). Mutation of human short tandem repeats. *Hum. Mol. Genet.*, **2**, 1123–1128.

WEBER JL, KWITEK AE AND MAY PE. (1990). Dinucleotide repeat polymorphisms at the D5S107, D5S108, D5S111, D5S117 and D5S118 loci. *Nucleic Acids Res.*, **18**, 4035.

WOOSTER R, CLETON-JANSEN A-M, COLLINS N, MANGION J, CORNELIS RS, COOPER CS, GUSTERSON BA, PONDER BAJ, VON DEIMLING A, WIESTLER OD, CORNELISSE CJ, DEVILEE P AND STRATTON MR. (1994). Instability of short tandem repeats (microsatellites) in human cancers. *Nature Genet.*, **6**, 152–156.

WRIGHT DK AND MANOS MM. (1990). Sample preparation from paraffin-embedded tissues. In *Protocols: A Guide to Methods and Applications*, Innis MA, Gelfand DH, Sninsky JJ and White TJ (eds) pp. 153–158. Academic Press: New York.

YOUNG RH, CLEMENT PB AND SCULLY RE. (1989). The ovary. In *Diagnostic Surgical Pathology*, Sternberg SS. (ed.) pp. 1655–1734. Raven Press: New York.